# Mineralocorticoid receptor antagonists for heart failure: systematic review and meta-analysis

**DOI:** 10.1186/s12872-016-0425-x

**Published:** 2016-12-01

**Authors:** Nicolas M. Berbenetz, Marko Mrkobrada

**Affiliations:** Department of Medicine, Western University, 339 Windermere Road, London, ON N6A 5A5 Canada

**Keywords:** Heart failure, Heart failure with reduced ejection fraction, Heart failure with preserved ejection fraction, Mineralocorticoid receptor antagonists, Systematic review

## Abstract

**Background:**

Mineralocorticoid receptor antagonists (MRAs) have been associated with improved patient outcomes in patients with heart failure with reduced ejection fraction (HFrEF) but not preserved ejection fraction (HFpEF). We conducted a systematic review and meta-analysis of selective and nonselective MRAs in HFrEF and HFpEF.

**Methods:**

We searched Cochrane Central Register of Controlled Trials, MEDLINE and EMBASE. We included randomized controlled trials (RCT) of MRAs in adults with HFpEF or HFrEF if they reported data on major adverse cardiac events or drug safety.

**Results:**

We identified 15 studies representing 16321 patients. MRAs were associated with a reduced risk of cardiovascular death (RR 0.81 [0.75–0.87], I^2^ 0%), all-cause mortality (RR 0.83 [0.77–0.88], I^2^ 0%), and cardiac hospitalizations (RR 0.80 [0.70–0.92], I^2^ 58.4%). However, an *a-priori* specified subgroup analysis demonstrated that these benefits were limited to HFrEF (cardiovascular death RR 0.79 [0.73–0.86], I^2^ 0%; all-cause mortality RR 0.81 [0.75–0.87], I^2^ 0%; cardiac hospitalizations RR 0.76 [0.64–0.90], I^2^ 68%), but not HFpEF (all-cause mortality RR 0.92 [0.79–1.08], I^2^ 0%; cardiac hospitalizations RR 0.91 [0.67–1.24], I^2^ 17%). MRAs increased the risk of hyperkalemia (RR 2.03 [1.78–2.31], I^2^ 0%). Nonselective MRAs, but not selective MRAs increased the risk of gynecomastia (RR 7.37 [4.42–12.30], I^2^ 0% vs. RR 0.74 [0.43–1.27], I^2^ 0%). Evidence was of moderate quality for cardiovascular death, all-cause mortality and cardiovascular hospitalizations; and high-quality for hyperkalemia and gynecomastia.

**Conclusions:**

MRAs reduce the risk of adverse cardiac events in HFrEF but not HFpEF. MRA use in HFpEF increases the risk of harm from hyperkalemia and gynecomastia. Selective MRAs are equally effective as nonselective MRAs, without a risk of gynecomastia.

**Electronic supplementary material:**

The online version of this article (doi:10.1186/s12872-016-0425-x) contains supplementary material, which is available to authorized users.

## Background

Heart failure (HF) has significant morbidity and is often a result of impaired left ventricular myocardial function [1]. HF with preserved ejection fraction (HFpEF) involves impaired myocardial function with normal left ventricle size and ejection fraction; in contrast, HF with reduced ejection fraction (HFrEF) involves an enlarged left ventricle size and reduced ejection fraction. Evidence-based HF treatment reduces morbidity and mortality in HFrEF [2]. HFpEF prevalence is rising due to an ageing population, however, there are no treatments which reduce morbidity and mortality [3]. Diagnosing HFpEF is often confounded by the occurrence of similar symptoms in patients with multiple medical comorbidities [3]. The most prevalent risk factor for HFpEF is hypertension [3]. Several RCTs have explored the benefits of β-blockers [4], ARBs [5], ACEi [6], and mineralocorticoid receptor antagonists (MRAs) [7] in HFpEF and identified trends towards reduced cardiovascular morbidity and mortality [8]. The lack of strong evidence in HFpEF treatment has led to considerable treatment variation [9].

MRAs can be selective (e.g., eplerenone) or nonselective (e.g., spironolactone). Eplerenone was synthesized through chemical modification of spironolactone in order to enhance binding of mineralocorticoid receptors while reducing off-target binding to progesterone or androgen receptors [10]. Eplerenone is associated with lower rates of impotence, gynecomastia or breast pain in comparison to spironolactone [11, 12].

MRAs found initial use in HF exacerbations as diuretics in patients’ refractory to combined ACEi and loop diuretic therapy [13]. However, spironolactone at doses with no significant diuretic effect was found to reduce cardiovascular mortality [14]. This effect was presumably due to a reduction in myocardial and vascular fibrosis [14]. This effect may arise from spironolactone blocking aldosterone’s ability to stimulate collagen synthesis at the myocardial level [15]. Spironolactone and eplerenone have demonstrated significant mortality benefit in HFrEF [11, 12]. In contrast, MRAs in HFpEF do not reduce all-cause mortality, however, they do reduce hospitalizations, improve quality of life, and improve echocardiographic measurements of diastolic function [16].

Chronically elevated aldosterone levels contribute towards structural changes in the heart which promote water retention, myocardial fibrosis, and increased arrhythmogenicity [17]. MRAs in HFpEF improved echocardiographic and biochemical measures of diastolic function [16, 18]. However, a large prospective RCT in HFpEF patients treated with spironolactone did not demonstrate a significant benefit in terms of cardiovascular outcomes [7].

### Objectives

Our objectives were to evaluate the risks and benefits of MRA usage in adults with HF. We were particularly interested in differences between selective and nonselective MRAs in HFpEF and HFrEF in terms of cardiovascular outcomes and adverse effects.

## Methods

Our systematic review and meta-analysis complies with the PRISMA statement [19].

### Eligibility criteria

We included randomized controlled trials (RCTs) of MRAs vs. placebo or standard therapy in adults (≥18 years old) with HFpEF or HFrEF. Included trials evaluated nonselective MRAs (e.g., canrenone, spironolactone), and selective MRAs (e.g., eplerenone, finerenone). Included trials contained at least one outcome of interest: mortality (all-cause or cardiovascular), cardiovascular hospitalizations, hyperkalemia, or gynecomastia.

### Literature search

We searched the Cochrane Central Register of Controlled Trials (*The Cochrane Library* Issue 1, 2016), MEDLINE (January 1995 to January 29, 2016), and EMBASE (January 1995 to January 29, 2016) for articles meeting our inclusion criteria. Our search strategy for Ovid MEDLINE and EMBASE is in Appendix 1 and our search strategy for the Cochrane Register of Controlled Trials is in Appendix 2. Our search did not have any language restrictions. We excluded reviews, editorials, and conferences but not unpublished studies or abstracts.

### Study selection

We entered the retrieved citations into Reference Manager (v12.0.3), and duplicate records were removed. One investigator (NB) screened citations for relevance based on their title and abstract. Both investigators reviewed the full text articles of relevant articles for study inclusion. Cohen’s kappa statistic was used to quantify chance-corrected agreement between the investigators. Disagreements on study inclusion were resolved through a consensus process of having a discussion between the two investigators.

### Data collection and analysis

Both investigators extracted data independently from included articles. We resolved disagreements during data extraction by consensus. If data were incomplete or unclear we attempted to contact trial authors. We extracted the following items from each study: population (type of heart failure, study size), intervention (MRA type), control (placebo, none, other), and outcomes (all-cause mortality, cardiovascular mortality, hospitalizations, hyperkalemia, and gynecomastia/breast pain). We used each study’s definition of these outcomes.

### Risk of bias

Our risk of bias assessment was completed using the Cochrane Risk of Bias Tool. It evaluates individual studies for several biases: selection, performance, detection, attrition and reporting. We evaluated the quality of evidence for each outcome using GRADE criteria [20], which evaluates an outcome across studies based on risk of bias, inconsistency, indirectness, imprecision and publication bias.

### Statistical analysis

We obtained the relative risk for each outcome from the original study and used RevMan (version 5.3.5) and R [21, 22] to analyze data and generate figures. We used the Mantel-Haenszel method with a 95% confidence interval, and a random effects model to pool results. We quantified statistical heterogeneity using the I^2^ statistic. We interpreted an I^2^ value of 0–25% as low heterogeneity, 25–50% as moderate heterogeneity, and >50% as high heterogeneity. *A priori* we established two hypotheses to explain potential heterogeneity: HF type (HFpEF and HFrEF), and MRA type (selective, or nonselective). We assessed for publication bias using funnel plots for each outcome.

## Results

### Trial selection

**Fig. 1:**
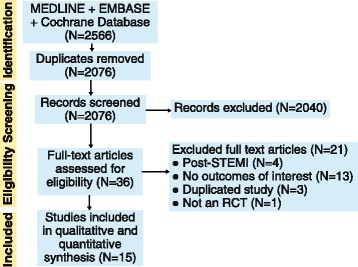
Study selection flow diagram. Overview of process used to identify studies for inclusion in the systematic review. Three databases (MEDLINE, EMBASE, Cochrane) were searched for relevant articles. After identification, studies were screened against our inclusion criteria. Included studies were used in our meta-analysis

We screened 2566 citations, and selected 36 for full text review. Of these, 15 articles [7, 11, 12, 18, 23–33] met our inclusion criteria and were included in our systematic review (see Fig. 1). Overall, there was excellent agreement on trial eligibility (Cohen’s kappa 94%). We excluded articles from the systematic review because of treatment in a non-HF setting (*N* = 4), lack of relevant outcomes (*N* = 13), study duplication (*N* = 3), and not an RCT design (*N* = 1).

### Trial characteristics

Table 1 reports the trial characteristics of the 15 RCTs containing 16321 patients. The patients had either HFpEF (*N* = 4027) or HFrEF (*N* = 12294) and the MRA treatment group was either nonselective, e.g., canrenone, spironolactone, *N* = 11 RCTs, 6678 patients; or selective, e.g., eplerenone, *N* = 4 RCTs, 9643 patients. Studies had an average length of follow-up of 15 months.

**Table 1:** Overview of trials meeting systematic review inclusion criteria

Author	Year	Population	Exp (N)	Cont (N)	Intervention	Drug dose	Follow-up (months)
Akbulut	2003	HFrEF, EF ≤ 35%, NYHA III	35	35	spironolactone	25 mg daily	3
Boccanelli	2009	HFrEF, EF ≤ 45%, NYHA II	215	223	canrenone	25 mg daily	12
Chan	2007	HFrEF, EF < 40%, NYHA I–III	23	25	spironolactone	25 mg daily	12
Cicoira	2002	HFrEF, EF ≤ 45%, NYHA III	54	52	spironolactone	25 mg daily	12
Deswal	2011	HFpEF, EF ≥ 50%, NYHA II–III	25	23	eplerenone	25 mg daily	6
Edelmann	2013	HFpEF, EF ≥ 50%, NYHA II–III	213	209	spironolactone	25 mg daily	12
Edwards	2009	HFpEF, CKD stage 2–3	56	56	spironolactone	25 mg daily	9
Zannad	2011	HFrEF, EF ≤ 35%, NYHA II	1364	1373	eplerenone	25–50 mg daily	21
Pitt	2003	MI + HFrEF, EF ≤ 40%	3319	3313	eplerenone	25–50 mg daily	16
Gao	2007	HFrEF, EF < 45%, NYHA II–IV	58	58	spironolactone	20 mg daily	6
Pitt	2013	HFrEF, EF ≤ 40%, CKD stage 2–3	63	65	spironolactone	25–50 mg daily	1
Pitt	1999	HFrEF, EF < 35%, NYHA III–IV	822	841	spironolactone	25–50 mg daily	24
Pitt	2014	HFpEF, EF ≥ 45%	1722	1723	spironolactone	15–45 mg daily	40
Udelson	2010	HFrEF, EF ≤ 35% NYHA II–III	117	109	eplerenone	50 mg daily	9
Vizzardi	2014	HFrEF, EF < 40%, NYHA I–II	65	65	spironolactone	25–100 mg daily	44

### Risk of bias within included trials

Table 2 reports the quality of included studies. Five trials had unclear or absent allocation concealment [23, 25, 26, 28, 30]. Two studies had inadequate blinding and were of single-blind design [23, 32]. Two large studies were terminated early due to meeting pre-defined benefit criteria [11, 33]. Another two studies did not use intention-to-treat analysis. Overall, loss-to-follow-up was low with a range of 0 to 6.6%.

**Table 2:** Risk of bias summary for each study included in the meta-analysis

Author	Year	Allocation concealment	Blinding	Intention to treat analysis	Loss to follow-up (%)	Early trial termination
Akbulut	2003	Unclear	No	Yes	0.0	No
Boccanelli	2009	Yes	Yes	Yes	6.2	No
Chan	2007	Unclear	Yes	Yes	0.0	No
Cicoira	2002	Unclear	Yes	Yes	6.6	No
Deswal	2011	Yes	Yes	No	4.3	No
Edelmann	2013	Yes	Yes	Yes	1.2	No
Edwards	2009	Unclear	Yes	No	2.7	No
Zannad	2011	Yes	Yes	Yes	1.2	Yes
Pitt	2003	Yes	Yes	Yes	0.3	No
Gao	2007	Yes	Yes	Yes	0.0	No
Pitt	2013	No (open label Aldactone)	Yes	Yes	0.0	No
Pitt	1999	Yes	Yes	Yes	0.0	Yes
Pitt	2014	Yes	Yes	Yes	3.8	No
Udelson	2010	Yes	Yes	Yes	0.0	No
Vizzardi	2014	Yes	No	Yes	0.0	No

### Results of meta-analysis

Table 3 reports a summary of findings. We included outcomes for cardiovascular death (7 RCTs), all-cause mortality (12 RCTs), cardiac hospitalization (10 RCTs), hyperkalemia (15 RCTs), and gynecomastia (*N* = 11 RCTs). Quality of evidence for cardiovascular death, all-cause mortality, and cardiac hospitalization were rated moderate; hyperkalemia and gynecomastia were rated high using GRADE guidelines [20]. For each outcome, HFrEF evidence was of high quality, but the quality of evidence for HFpEF was of moderate quality for all-cause mortality, cardiovascular death, and cardiac hospitalizations.

**Table 3:** Summary of findings for the effect of mineralocorticoid receptor antagonists in treating Heart Failure

Outcome	№ of participants (studies)	Quality of the evidence (GRADE)	Relative effect (95% CI)	Anticipated absolute effects	
				Risk with placebo	Risk difference with MRA
Cardiovascular death	15115 (7 RCTs)	⨁⨁⨁ MODERATE^a^	RR 0.81 (0.75 to 0.87)	155 per 1000	29 fewer per 1000 (39 fewer to 20 fewer)
Cardiovascular death - rEF	11670 (6 RCTs)	⨁⨁⨁⨁ HIGH	RR 0.79 (0.73 to 0.86)	171 per 1000	36 fewer per 1000 (46 fewer to 24 fewer)
Cardiovascular death - pEF	3445 (1 RCT)	⨁⨁⨁ MODERATE^b^	RR 0.91 (0.74 to 1.11)	102 per 1000	9 fewer per 1000 (27 fewer to 11 more)
All cause mortality	15919 (12 RCTs)	⨁⨁⨁ MODERATE^c^	RR 0.83 (0.77 to 0.88)	182 per 1000	31 fewer per 1000 (42 fewer to 22 fewer)
All cause mortality - rEF	11892 (8 RCTs)	⨁⨁⨁⨁ HIGH	RR 0.81 (0.75 to 0.87)	197 per 1000	38 fewer per 1000 (49 fewer to 26 fewer)
All cause mortality - pEF	4027 (4 RCTs)	⨁⨁⨁ MODERATE^d^	RR 0.92 (0.79 to 1.08)	136 per 1000	11 fewer per 1000 (29 fewer to 11 more)
Cardiac hospitalization	15669 (10 RCTs)	⨁⨁⨁ MODERATE^d^	RR 0.80 (0.70 to 0.92)	217 per 1000	43 fewer per 1000 (65 fewer to 17 fewer)
Cardiac hospitalization - rEF	11754 (7 RCTs)	⨁⨁⨁ MODERATE^d^	RR 0.76 (0.64 to 0.90)	245 per 1000	59 fewer per 1000 (88 fewer to 24 fewer)
Cardiac hospitalization - pEF	3915 (3 RCTs)	⨁⨁⨁ MODERATE^d^	RR 0.91 (0.67 to 1.24)	134 per 1000	12 fewer per 1000 (44 fewer to 32 more)
Hyperkalemia	16321 (15 RCTs)	⨁⨁⨁⨁ HIGH	RR 2.03 (1.78 to 2.31)	37 per 1000	39 more per 1000 (29 more to 49 more)
Gynecomastia or breast pain - nonselective	6432 (8 RCTs)	⨁⨁⨁⨁ HIGH	RR 7.37 (4.42 to 12.30)	5 per 1000	30 more per 1000 (16 more to 53 more)
Gynecomastia or breast pain - selective	9417 (3 RCTs)	⨁⨁⨁⨁ HIGH	RR 0.74 (0.43 to 1.27)	7 per 1000	2 fewer per 1000 (4 fewer to 2 more)

*CI* Confidence interval, *RR* Risk ratio

^a^High quality of evidence for HFrEF, single study for HFpEF

^b^Single trial with confidence interval which crossed unity

^c^High quality of evidence for HFrEF, moderate quality evidence for HFpEF

^d^Confidence interval of data crossed unity

**Fig. 2:**
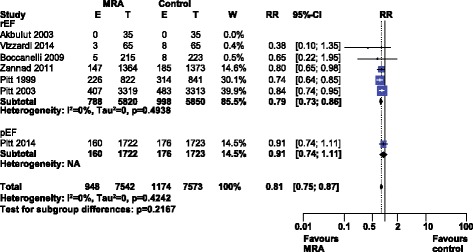
Forest plot of cardiovascular death with MRA use in HF. Seven trials reported cardiovascular death rates when using MRAs in HF compared to control. Our Forest plot has been subdivided according to HF type

Meta-analysis of cardiovascular death (see Fig. 2) revealed a significant risk reduction, RR 0.81 [0.75–0.87], I^2^ 0% (low heterogeneity). Our analysis of cardiovascular death by HF type indicated only a single trial of HFpEF (TOPCAT) which had no significant reduction in cardiovascular death [7]. Using either selective or nonselective MRA had a similar reduction in cardiovascular death (Additional file 1: Figure S1).

**Fig. 3:**
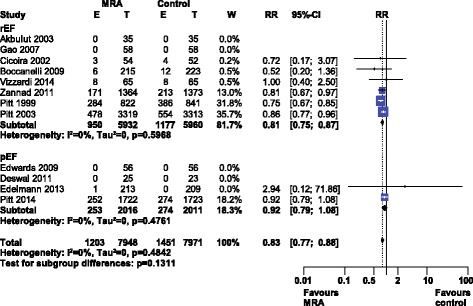
Forest plot of all-cause mortality with MRA use in HF. Twelve trials reported all-cause mortality rates with MRA use in HF compared to control. Our Forest plot has been subdivided according to HF type

Meta-analysis of all-cause mortality (see Fig. 3) revealed a significant risk reduction, RR 0.83 [0.77–0.88], I^2^ 0% (low heterogeneity). HF type subgroups indicated the benefit was limited to HFrEF. Use of either a selective or nonselective MRA had a similar reduction in all-cause mortality (Additional file 2: Figure S2).

**Fig. 4:**
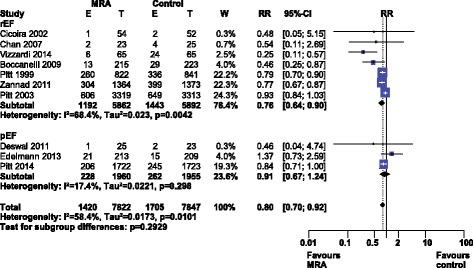
Forest plot of cardiovascular hospitalizations with MRA use in HF. Ten trials reported cardiovascular hospitalization rates with MRA use in HF compared to control. Our Forest plot has been subdivided according to HF type

Meta-analysis of cardiac hospitalizations (see Fig. 4) revealed a significant risk reduction, RR 0.80 [0.70–0.92], I^2^ 58.4% (high heterogeneity). Our *a priori* subgroup analysis partially explained the heterogeneity within this outcome, as a significant reduction in cardiac hospitalizations was found in the HFrEF and nonselective MRA subgroups (Additional file 3: Figure S3).

**Fig. 5:**
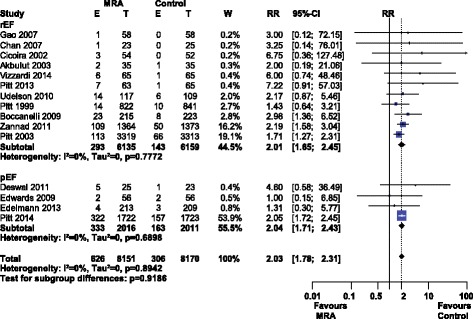
Forest plot of hyperkalemia with MRA use in HF. Fifteen trials reported hyperkalemia rates with MRA use in HF compared to control. Our Forest plot has been subdivided according to HF type

Hyperkalemia was significantly more common with MRA use, RR 2.03 [1.78–2.31], I^2^ 0% (low heterogeneity), see Fig. 5. Subgroup analysis by MRA or HF type did not significantly influence the rate of hyperkalemia (Additional file 4: Figure S4).

**Fig. 6:**
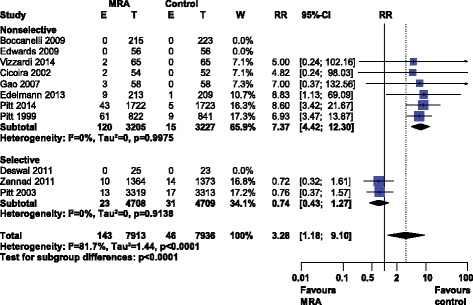
Forest plot of gynecomastia with MRA use in HF. Eleven trials reported gynecomastia rates with MRA use in HF compared to control. Our Forest plot has been subdivided according to MRA type

Gynecomastia was significantly more common with MRA use, RR 3.28 [1.18–9.10], I^2^ 81.7% (high heterogeneity), see Fig. 6. MRA type explained this heterogeneity as selective MRAs did not produce significant amounts of gynecomastia (RR 0.74 [0.43–1.27], I^2^ 0%) while nonselective MRAs did (RR 7.37 [4.42–12.30], I^2^ 0%).

Our analysis of funnel plots for each outcome except gynecomastia revealed no significant asymmetry (Additional file 5: Figure S5, Additional file 6: Figure S6, Additional file 7: Figure S7, Additional file 8: Figure S8 and Additional file 9: Figure S9) and suggested the absence of publication bias. Two MRA subgroups within the funnel plot for gynecomastia explained the asymmetry of the plot (Additional file 9: Figure S9).

## Discussion

### Summary of evidence

15 trials evaluated the use of MRAs compared to placebo or no treatment for HF. MRA use in patients with heart failure was associated with a significant reduction in adverse cardiovascular outcomes: cardiovascular death (RR 0.81 [0.75–0.87], I^2^ 0%), all-cause mortality (RR 0.83 [0.77–0.88], I^2^ 0%), and cardiac hospitalizations (RR 0.80 [0.70–0.92], I^2^ 58.4%). *Our a priori* specified subgroup analysis demonstrated that the benefits of MRAs are limited to HFrEF. Both selective and nonselective MRAs increase the risk of hyperkalemia (RR 2.03 [1.78–2.31], I^2^ 0%), but gynecomastia is limited to nonselective MRAs (nonselective MRAs RR 7.37 [4.42–12.30], I^2^ 0% vs. selective MRAs RR 0.74 [0.43–1.27], I^2^ 0%RR 7.37 [4.42–12.30).

### Strengths and limitations

Our systematic review has strengths including adherence to PRISMA reporting guidelines. In addition, our conclusions are based on evidence of moderate and high quality (GRADE). HFpEF evidence was of moderate quality, and HFrEF evidence was of high quality for cardiovascular death and all-cause mortality. The quality of evidence for cardiovascular death and all-cause mortality was reduced due the evidence for MRA use in HFpEF being limited to a single trial with large effect size [7], and several smaller trials with confidence intervals crossing unity [18, 27, 28]. For cardiovascular hospitalizations, the quality of evidence was reduced by confidence intervals in HFpEF and HFrEF studies crossing unity [7, 33]. Evidence for hyperkalemia and gynecomastia with MRA usage was of high quality. Overall, the evidence supporting MRA use in HFrEF is based on a larger number of trials with significant effect sizes for reducing adverse cardiac events. In contrast, the evidence for MRA use in HFpEF is based on a smaller number of trials, only one of which had a significant reduction in cardiovascular hospitalizations but no other adverse cardiac events [7]. Finally, our conclusions supporting MRA usage in HFrEF align with current American Heart Association guidelines which recommend MRAs for patients with HFrEF and NYHA class II-IV symptoms or following acute MI complicated by HF and EF ≤ 40% [1].

### Implications

Current guidelines suggest MRAs are useful in treating HFrEF and acute MI complicated by HF [1, 34]. We demonstrate that treatment of HFpEF with MRAs does not reduce adverse cardiac events. However, MRAs do cause harm from hyperkalemia (NNH 26 [20–34]) and gynecomastia (e.g., nonselective MRA, NNH 33 [19–63]). Selective MRAs offer a slight advantage in terms of no significant gynecomastia while having equivalent reductions in adverse cardiac outcomes. We suggest continued usage of MRAs in HFrEF, where there is a significant reduction in adverse cardiac outcomes, e.g., cardiovascular death (NNT 34 [26–50]), or all-cause mortality (NNT 32 [24–45]). We suggest that MRAs be avoided in HFpEF as they do not reduce adverse cardiovascular outcomes.

## Conclusions

Our systematic review provides evidence that MRAs should not be used in HFpEF. MRA usage in HFpEF provides a risk of hyperkalemia and/or gynecomastia without reducing adverse cardiac events. In contrast, MRA usage in HFrEF significantly reduces adverse cardiac events.

## Appendix 1

**Table 4:** Ovid MEDLINE and EMBASE search strategy

1	Exp heart failure/	376335/94836
2	Exp Cardiomyopathy/	113241/78224
3	Exp Ventricular dysfunction/	13819/28965
4	((heart or cardiac or myocardial) adj2 (failure or decompensation)).ti,ab	211838/130125
5	((congestive or chronic) adj2 heart failure).ti,ab	67554/45613
6	((ventric$) adj2 (failure or insufficien$ or dysfunction$ or function$)).ti,ab	82523/54772
7	((Reduced or preserved) adj2 "ejection fraction").ti,ab.	6293/2856
8	(HFpEF or HFrEF).mp	2216/776
9	((diastol$ or systol$) adj2 ((failure or dysfunction$ or function$)).ti,ab	48968/27523
10	Or/1-9	538115/275995
11	(animal$ not (human$ and animal$)).sh,hw.	3987846/4137327
12	10 NOT 11	501141/241626
13	exp Aldosterone Antagonist/	32899/8124
14	(eplerenone or inspra or spironolactone or aldactone or aldo$ or mineralocorticoid receptor antagon$ or canren$ or fineren$). ti,ab	134477/63667
15	(aldosterone adj2 antagon$). ti,ab	7857/2066
16	Or/13-15	134563/63667
17	16 NOT 11	111915/46794
18	12 and 17	20116/4880
19	(1995* or 1996* or 1997* or 1998* or 1999* or 2000* or 2001* or 2002* or 2003* or 2004* or 2005* or 2006* or 2007* or 2008* or 2009* or 2010* or 2011* or 2012* or 2013* or 2014* or 2015* or 2016*).dd	21741062/0
20	(1995* or 1996* or 1997* or 1998* or 1999* or 2000* or 2001* or 2002* or 2003* or 2004* or 2005* or 2006* or 2007* or 2008* or 2009* or 2010* or 2011* or 2012* or 2013* or 2014* or 2015* or 2016*).ed	0/16309819
21	19 or 20	21741062/16309819
22	18 AND 21	17787/3735
23	(book or conference paper or editorial or letter or review).pt. not exp randomized controlled trial/	4298634/3319843
24	(random sampl$ or random digit$ or random effect$ or random survey or random regression).ti,ab. not exp randomized controlled trial/	69917/56001
25	(random$ or placebo$ or single blind$ or double blind$ or triple blind$).ti,ab.	1177770/884750
26	25 not (23 or 24 or 11)	908857/648772
27	22 and 26	1802/503 = 2305
28	Remove duplicates from 27	1372/502 = 1874

(Search performed January 29, 2016)

## Appendix 2

**Table 5:** Cochrane database search strategy

1	exp heart failure	1412
2	exp Cardiomyopathy	133
3	exp Ventricular dysfunction	225
4	((heart or cardiac or myocardial) near/2 (failure or decompensation)):ti,ab,kw	14941
5	((congest* or chronic) near/2 "heart failure"):ti,ab,kw	6158
6	(ventric* near (fail* or insufficien* or dysfunction* or function*)):ti,ab,kw	9067
7	((diastol* or systol*) near/2 (failure or dysfunction* or function*)):ti, ab, kw	2866
8	((Reduced or preserved) near/2 (ejection or fraction or “EF”)):ti, ab, kw	532
9	(“HFpEF” or “HFrEF”):ti, ab, kw	161
10	#1 or #2 or #3 or #4 or #5 or #6 or #7 or #8 or #9	21983
11	exp Aldosterone Antagonist*	44
12	(mineralocorticoid receptor antagonist*):ti,ab,kw	486
13	(eplerenone or inspra or spironolactone or aldactone or canren* or fineren*):ti,ab,kw	1309
14	Aldosterone near/2 antagon*	393
15	#11 or #12 or #13	1661
16	#10 and #15	692

Updated Jan-29-2016

## Additional files

Additional file 1: Figure S1. Forest plot of cardiovascular death in HF with MRA use by MRA type. (EPS 2561 kb)
Additional file 2: Figure S2. Forest plot of all-cause mortality in HF with MRA use by MRA type. (EPS 2763 kb)
Additional file 3: Figure S3. Forest plot of cardiovascular hospitalization with MRA use by MRA type. (EPS 2740 kb)
Additional file 4: Figure S4. Forest plot of hyperkalemia with MRA use by MRA type. (EPS 2919 kb)
Additional file 5: Figure S5. Funnel plot of cardiovascular death with MRA use. (EPS 737 kb)
Additional file 6: Figure S6. Funnel plot of all-cause mortality with MRA use. (EPS 736 kb)
Additional file 7: Figure S7. Funnel plot of cardiovascular hospitalizations with MRA use. (EPS 710 kb)
Additional file 8: Figure S8. Funnel plot of hyperkalemia with MRA use. (EPS 736 kb)
Additional file 9: Figure S9. Funnel plot of gynecomastia with MRA use. (EPS 743 kb)

## Abbreviations

HF: Heart failure
HFpEF: Heart failure with preserved ejection fraction
HFrEF: Heart failure with reduced ejection fraction
MI: Myocardial infarction
MRA: Mineralocorticoid receptor antagonist
NNH: Number needed to harm
NNT: Number needed to treat
NYHA: New York Heart Association
RCT: Randomized controlled trial
RR: Relative risk
